# Evaluating the Diagnostic Value of Absolute Eosinopenia in Culture-Positive Enteric Fever: A Retrospective Case-Control Study

**DOI:** 10.7759/cureus.88029

**Published:** 2025-07-15

**Authors:** Gyanamitra Panigrahi, Kunalsen Jagatdeo, Soumayan Mondal, Manaranjan Malik, Medichetti Harish Kumar, Dipti Pattnaik, Shubhransu Patro, Sidharth S Pattnaik

**Affiliations:** 1 Internal Medicine, Kalinga Institute of Medical Sciences, Bhubaneswar, IND; 2 Microbiology, Kalinga Institute of Medical Sciences, Bhubaneswar, IND

**Keywords:** diagnostic markers, enteric fever, eosinopenia, typhidot, widal test

## Abstract

Background

Enteric fever continues to pose a diagnostic challenge in endemic regions due to the limited availability and delayed results of blood cultures and the poor specificity of serological tests. Absolute eosinophil count (AEC = 0 cells/mm³), a simple and low-cost hematological marker, may offer early diagnostic utility, especially in resource-constrained settings.

Objective

To evaluate the diagnostic performance of absolute eosinopenia in detecting culture-confirmed enteric fever and compare it with commonly used serological tests such as Widal and Typhidot.

Methods

This retrospective case-control study was conducted at a tertiary care teaching hospital in eastern India between August 2023 and June 2024. Culture-positive patients with *Salmonella Typhi* or *S. Paratyphi* were enrolled as cases, while febrile patients with confirmed non-enteric diagnoses served as controls. Data were extracted from electronic health records. A total of 159 patients (79 cases and 80 controls) were included. Sample size was calculated using prior eosinopenia proportions (59% vs. 13%), with α = 0.05 and 80% power. Absolute eosinophil counts were obtained using an automated hematology analyzer, and diagnostic accuracy was compared to serological testing.

Results

Absolute eosinopenia was observed in 46.8% of enteric fever cases and 8.75% of controls. It showed a sensitivity of 45.6% (95% CI: 34.3-57.3) and a specificity of 91.3% (95% CI: 82.7-96.4), with a positive predictive value of 84.6% and a negative predictive value of 61.6%. The area under the receiver operating characteristic (ROC) curve for AEC was 0.68 (95% CI: 0.60-0.76), demonstrating moderate diagnostic accuracy. These results were comparable or superior to commonly used serological tests.

Conclusion

Absolute eosinopenia demonstrates high specificity and moderate overall diagnostic accuracy for enteric fever. It may serve as a rapid, adjunctive marker to support clinical suspicion in endemic, resource-limited settings - particularly where blood culture or reliable serological testing is unavailable. However, it should be interpreted alongside leukocyte counts and clinical features rather than used as a standalone diagnostic criterion.

Limitations

As a retrospective, single-center study, findings may be influenced by selection bias and may not be generalizable across broader populations. The timing of eosinophil count measurement relative to symptom onset was not standardized. Further prospective studies are needed to validate these findings.

## Introduction

Global burden estimates underscore the ongoing importance of early diagnosis and treatment. A systematic review estimated that low and middle income countries (LMICs) accounted for over 11.9 million cases and 129,000 deaths due to typhoid fever in 2010, with substantial underreporting likely [1]. The Global Burden of Disease Study 2017 similarly identified South Asia as bearing the highest burden, with nearly 11 million cases globally in that year [2].

Enteric fever, caused by Salmonella Typhi and Salmonella Paratyphi, remains a major global health concern, predominantly in regions with limited sanitation and inadequate water supply. The bacteria are transmitted mainly through contaminated food or water via the fecal-oral route, after which they invade the ileal lymphatic system and disseminate throughout the reticuloendothelial system, resulting in multi-organ involvement [3,4].

Epidemiological data suggest that enteric fever affects over 21 million people each year, with an estimated 200,000 annual deaths worldwide. The highest incidence rates are found in South-Central Asia, Southeast Asia, and parts of Africa [5]. In these regions, enteric fever predominantly impacts children and young adults, with outbreaks frequently associated with poor sanitation and inadequate water supply. The disease manifests with a range of symptoms, including prolonged fever, abdominal pain, and gastrointestinal distress. Severe cases can lead to complications such as intestinal perforation and septicemia, necessitating prompt and effective treatment.

Although blood culture remains the gold standard for diagnosing enteric fever, its sensitivity varies from 40-70%, heavily influenced by prior antibiotic use, timing of sample collection, and bacterial load. Bone marrow aspirate cultures, though more sensitive, are invasive and not routinely performed in many settings [6,7]. Serological tests such as the Widal test and Typhidot (IgM and IgG) are cost-effective but have variable sensitivity and low specificity, resulting in potential misdiagnoses [8].

Hematological abnormalities, including anemia, elevated erythrocyte sedimentation rate (ESR), and thrombocytopenia, often accompany enteric fever. Leukopenia, while considered a characteristic finding, is present in only about 20-25% of cases, thus limiting its diagnostic utility [9]. Eosinopenia, characterized by a reduction in eosinophil count, has been noted in several bacterial infections including sepsis, pneumonia, and urinary tract infections, and may also be present in a significant proportion of enteric fever cases [10,11]. Specifically, absolute eosinopenia (defined as an eosinophil count of 0 cells/mm³) has been observed in a large proportion of enteric fever cases in some studies [12,13]. The pathophysiology underlying this observation includes the arrest of myeloid maturation, reduced levels of erythroblasts and megakaryocytes, and increased phagocytosis in the bone marrow. Additionally, eosinophils are rapidly sequestered in the spleen in response to C5a and fibrin, leading to their decreased levels in peripheral circulation [12].

Given the time-sensitive need for accurate diagnosis and the limitations of current methods, eosinopenia may serve as an additional rapid, cost-effective marker for enteric fever, especially in resource-limited settings. This study evaluates the diagnostic utility of eosinopenia - particularly absolute eosinopenia - in comparison to conventional serological tests (Typhidot and Widal) and aims to establish its potential role as an early indicator of enteric fever.

## Materials and methods

Study design and ethics approval

This retrospective case-control study was conducted over a one-year period, from August 2023 to June 2024, at the Kalinga Institute of Medical Sciences, Bhubaneswar. Ethical approval was obtained from the Institutional Review Board (IRB Approval No: KIIT/KIMS/IEC/1902/2024). Informed consent was waived under retrospective study exemption criteria, as approved by the IEC, in accordance with institutional and national ethical guidelines.

Participants

The study included two groups: culture-confirmed enteric fever cases and febrile controls. The case group comprised all patients with blood culture-positive results for Salmonella Typhi or Salmonella Paratyphi. The control group included patients admitted during the same time period with acute febrile illness who had blood cultures negative for Salmonella Typhi and Paratyphi. These controls consisted of patients with clinically diagnosed viral infections, malaria, or undifferentiated febrile illnesses that did not meet the criteria for enteric fever.

Inclusion criteria for the case group were all individuals with blood culture-confirmed enteric fever. Exclusion criteria for both groups included patients with hematological malignancies, bronchial asthma, atopic dermatitis, filarial disease, active alcohol use, or those receiving corticosteroids or immunosuppressive therapy.

Data collection

Clinical and laboratory data were retrieved from electronic health records using a structured abstraction protocol. Identifiable patient data were not recorded, ensuring full confidentiality. Complete blood count (CBC), including absolute eosinophil count (AEC), was obtained at initial presentation, prior to the initiation of antibiotic therapy. Measurements were performed using the Sysmex XN-1000 Automated Hematology Analyzer (Sysmex Corporation, Kobe, Japan). Absolute eosinopenia was defined as an eosinophil count of 0 cells/μL. Blood cultures were performed using the BACTEC and BACT/ALERT 3D automated blood culture systems, and bacterial identification was confirmed using the VITEK system (BioMérieux SA, Marcy l’Etoile, France). Typhidot testing was conducted using a rapid immunochromatographic assay that detects IgM and IgG antibodies against Salmonella Typhi and Paratyphi, with results available within 30 minutes.

Sample size calculation

The sample size was calculated using the statsmodels module in Python, based on proportions reported by Ishaq et al. (2020), which identified absolute eosinopenia in 59% of enteric fever cases and 13% of controls. The calculation assumed a two-sided Chi-square test, alpha of 0.05, power of 80%, and a 1:1 case-to-control ratio. This yielded a minimum required sample of 60 participants per group (total N = 120). Our study enrolled 159 participants (79 culture-confirmed cases and 80 febrile controls), exceeding the required sample and ensuring adequate statistical power for diagnostic comparisons.

Statistical analysis

Statistical analysis was conducted using R version 4.3.3 (R Foundation for Statistical Computing, Vienna, Austria). Continuous variables were expressed as mean and standard deviation or median and interquartile range, depending on distribution. Categorical variables were summarized using frequencies and percentages. Group comparisons were performed using the independent t-test or Mann-Whitney U test for continuous variables, and the Chi-square test for categorical data.

Diagnostic performance of absolute eosinopenia and Typhidot was evaluated by calculating sensitivity, specificity, positive predictive value (PPV), and negative predictive value (NPV), each reported with 95% confidence intervals. Blood culture was considered the reference gold standard. Receiver operating characteristic (ROC) curve analysis was used to assess diagnostic performance, and DeLong’s test was used to compare the areas under the curve (AUCs) for eosinopenia and Typhidot. Agreement between diagnostic modalities was assessed using Cohen’s Kappa statistic. McNemar’s test was performed to compare discordant paired proportions between the diagnostic methods.

## Results

Demographic and hematological characteristics

A total of 159 patients were included in the study, comprising 79 culture-positive enteric fever cases and 80 febrile controls. The mean age across groups was 31.07 years (SD = 11.01), and the overall male predominance was 75.47%. Baseline demographic and hematological parameters are presented in Table 1.

**Table 1:** Baseline Characteristics and Hematological Parameters in Enteric Fever and Control Groups Comparison of demographic and hematological characteristics between enteric fever cases and controls. Parameters include age, total leukocyte count (TLC), eosinophil count, and absolute eosinophil count (AEC). Values are presented as mean ± standard deviation (SD), median, and minimum-maximum ranges, standard error (SE).

Variable	Group	N	Mean	SD	Median	Min	Max	SE
Age (years)	Cases	79	32.18	13.64	28.00	18	88	1.54
	Controls	80	29.98	7.50	29.50	19	45	0.84
TLC (cells/µL)	Cases	79	6451.01	2821.88	6010.00	1430	15600	317.49
	Controls	80	7975.81	2650.91	7475.00	3070	14478	296.38
Eosinophils (%)	Cases	79	0.60	1.26	0.10	0	9	0.14
	Controls	80	1.12	0.58	1.20	0	2	0.06
AEC (cells/µL)	Cases	79	41.16	80.59	8.97	0	504	9.07
	Controls	80	87.50	54.18	87.75	0	260.6	6.06

Culture-positive cases had significantly lower total leukocyte counts compared to controls (p = 0.014), along with significantly lower eosinophil counts (p = 0.0012). Absolute eosinopenia (AEC = 0 cells/μL) was observed in 46.8% of cases, with a significantly lower mean AEC in cases than controls (p < 0.001).

Serological test outcomes

The distribution of Widal and Typhidot test results among cases and controls is shown in Table 2. Typhidot was performed in 65 culture-positive cases and all 80 controls. It was positive in 28 out of 65 cases (43.08%) and in five out of 80 controls (6.25%), a statistically significant difference (p < 0.0001). Widal testing was performed in only nine of the 79 culture-positive cases and all 80 controls. Among the tested individuals, four of nine cases (44.44%) and seven of 80 controls (8.75%) were Widal positive (p = 0.0107). Due to the limited number of case samples, Widal test results are reported for completeness but were not used in interpretation or diagnostic comparisons.

**Table 2:** Distribution of Diagnostic Test Results and Patient Demographics Gender distribution and diagnostic test results (Widal and Typhidot) in cases and controls. Percentages reflect the proportion of individuals with positive and negative results for each test. Note: Widal test was performed in only nine out of 79 culture-positive cases due to inconsistent availability; hence, Widal data are included for completeness but were not used in key diagnostic comparisons or interpretations.

Variable	Group	Total Size(N)	Levels	Frequency	Percentage (%)
Gender	Cases	79	Female	18	22.78
			Male	61	77.22
	Controls	80	Female	21	26.25
			Male	59	73.75
Widal	Cases	9	Negative	5	55.56
			Positive	4	44.44
	Controls	80	Negative	73	91.25
			Positive	7	8.75
Typhidot	Cases	65	Negative	37	56.92
			Positive	28	43.08
	Controls	80	Negative	75	93.75
			Positive	5	6.25

Diagnostic accuracy of eosinopenia, Typhidot, and Widal

Table 3 summarizes the diagnostic performance of absolute eosinopenia and Typhidot, along with Widal (not used in interpretation). Absolute eosinopenia demonstrated a sensitivity of 45.6% (95% CI: 35.0-56.5), specificity of 91.2% (95% CI: 83.0-95.7), PPV of 83.7% (95% CI: 70.0-91.9), and NPV of 62.9% (95% CI: 53.9-71.2). Typhidot showed a sensitivity of 35.4% (95% CI: 25.8-46.4), specificity of 93.8% (95% CI: 86.2-97.3), PPV of 84.8% (95% CI: 69.1-93.3), and NPV of 59.5% (95% CI: 50.8-67.7). Widal yielded a sensitivity of 44.4% (95% CI: 18.9-73.3) and specificity of 91.2% (95% CI: 83.0-95.7), but its reliability is limited due to the small sample size in the case group. It was not used in statistical interpretation or conclusions.

**Table 3:** Sensitivity, Specificity, PPV, and NPV of Absolute Eosinopenia, Widal, and Typhidot Tests Performance metrics for diagnostic tests in enteric fever. Sensitivity, specificity, positive predictive value (PPV), and negative predictive value (NPV) for absolute eosinopenia, Widal, and Typhidot tests are compared.

Test	Sensitivity (%)	Specificity (%)	PPV (%)	NPV (%)
Absolute Eosinopenia	45.57	91.25	83.72	62.93
Typhidot	35.44	93.74	84.85	59.52
Widal	44.44	91.25	36.36	93.59

Receiver operating characteristic (ROC) analysis

**Figure 1:**
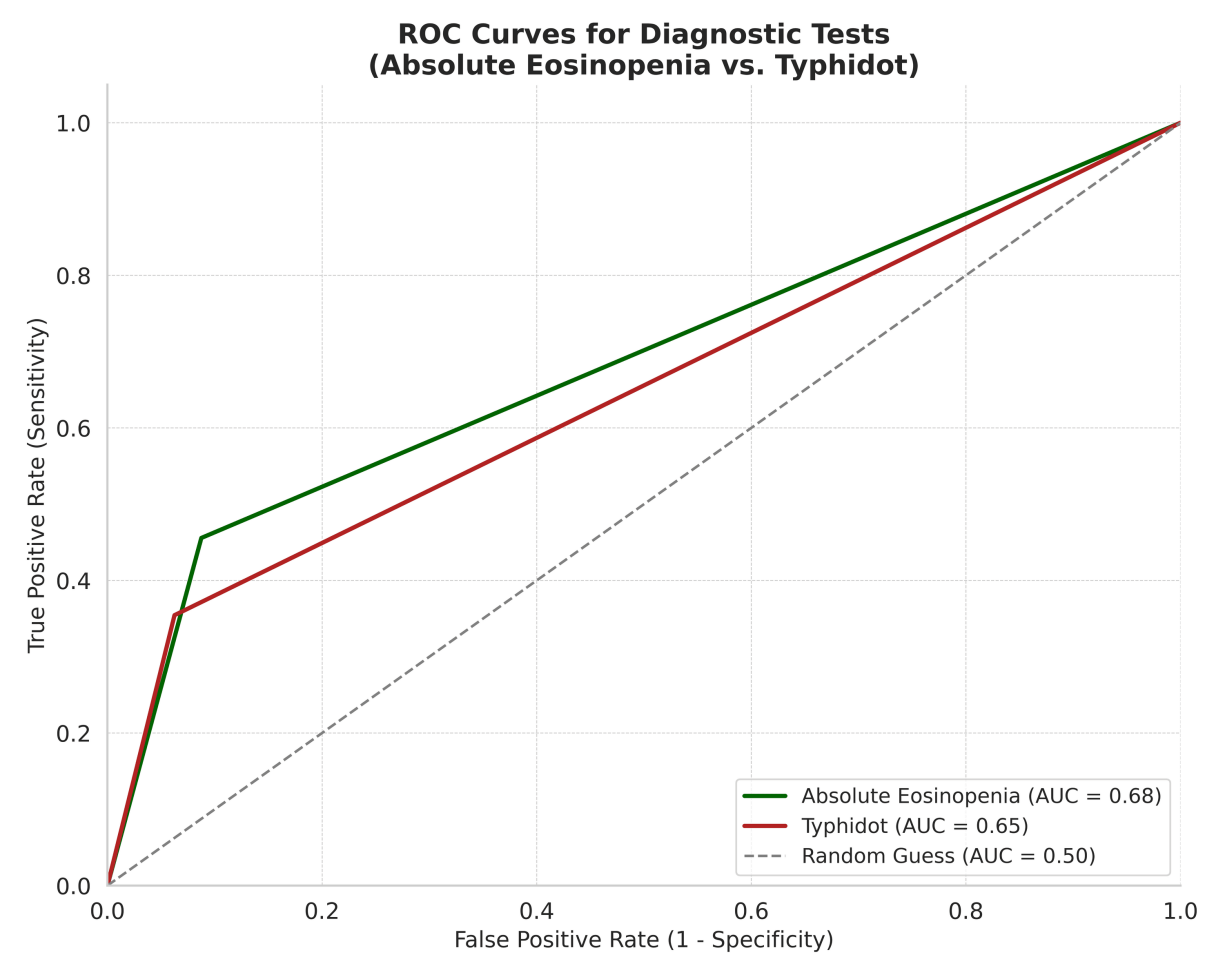
Receiver Operating Characteristic (ROC) Curves for Absolute Eosinopenia and Typhidot This chart compares the ROC curves of absolute eosinopenia and Typhidot tests, illustrating their relative diagnostic strengths. Area under the curve (AUC) values help determine which test offers better discrimination between cases and controls.

The ROC analysis comparing absolute eosinopenia and Typhidot is shown in Figure 1. Absolute eosinopenia yielded an AUC of 0.68 (95% CI: 0.60-0.76), indicating moderate diagnostic accuracy. Typhidot had a lower AUC of 0.62 (95% CI: 0.53-0.71). McNemar’s test demonstrated a significant difference in diagnostic classification between eosinopenia and Typhidot (p < 0.0001), suggesting these two tests are not interchangeable. Cohen’s Kappa coefficient for agreement between eosinopenia and Widal was 0.602 (p = 0.014), indicating moderate agreement. DeLong’s test found no statistically significant difference between the AUCs of eosinopenia and Typhidot (p = 0.934). Widal was excluded from the ROC analysis due to limited case numbers, which would produce unstable estimates.

## Discussion

Enteric fever remains a major global public health challenge, affecting over 21 million individuals annually, with an estimated 200,000 deaths worldwide [14]. This study aimed to evaluate the diagnostic performance of absolute eosinopenia, Typhidot, and Widal in identifying culture-positive enteric fever cases, contributing new insights to the diagnostic landscape. Given the limitations of blood culture (variable sensitivity) and serological tests (inconsistent specificity), alternative markers such as eosinopenia could serve as valuable adjuncts to improve early detection. Compared to the traditional Widal test, eosinopenia demonstrated higher sensitivity, specificity, PPV, and NPV, making it a more accurate and reliable diagnostic marker [15].

Our demographic analysis revealed a predominantly male population (75.47%) with a mean age of 31.07 years, consistent with previous regional epidemiological findings [14]. Hematological parameters showed significantly lower leukocyte and eosinophil counts in culture-positive cases compared to controls (p = 0.014 and p = 0.0012, respectively), reinforcing the diagnostic value of eosinopenia. These results align with those reported by Etouke et al. [16], who demonstrated similar eosinophil suppression in patients with enteric fever. Absolute eosinopenia (AEC = 0 cells/µL) was observed in 46.8% of our culture-confirmed cases, comparable to other studies showing eosinopenia in 42-81% of patients [12,17,18]. These data support its clinical utility as a rapid screening clue when culture results are pending or unavailable. Kapoor et al. also reported high specificity using eosinopenia thresholds in combination with leukocyte counts, supporting its role as a rule-in test [17].

Comparing diagnostic accuracy

Absolute eosinopenia in our study demonstrated a specificity of 91.25% and a sensitivity of 45.57%, making it a highly specific but moderately sensitive marker for enteric fever. These findings are consistent with previous research, including Lokhandwala et al. [13], who observed similar diagnostic utility. Typhidot showed the highest specificity (93.74%) but lower sensitivity (35.44%), while Widal demonstrated moderate performance (sensitivity 44.44%, specificity 91.25%), consistent with historical trends [19]. Given its moderate sensitivity, eosinopenia should complement, not replace, other diagnostic methods.

Enhanced predictive accuracy with multi-parameter approaches

Kapoor et al. demonstrated that combining AEC <14/mm³ with total leukocyte count <8×10⁹/L yields a 95.6% diagnostic accuracy with only 4.4% misclassification [17]. This suggests that eosinopenia is best interpreted as part of a hematological pattern, especially in endemic settings with limited resources.

Timing and broader diagnostic context

In this study, eosinophil counts were recorded at the time of admission, often during the early febrile phase. While we did not assess dynamic changes over time, previous studies suggest eosinopenia develops early in bacterial illnesses-often preceding leukocytosis or culture positivity [9]. Mésinèle et al. found that an eosinophil count <0.01 g/L was a strong early indicator of bacterial infection in elderly inpatients, with high specificity [20]. However, as Zhou et al. showed in COVID-19 cohorts, eosinopenia is not exclusive to enteric fever and may occur in viral or mixed infections, emphasizing the need for clinical correlation [21]. The false positives observed in 6.25% of controls likely reflect nonspecific eosinopenia due to other undiagnosed bacterial or systemic conditions. Conversely, false negatives could represent patients with partial bone marrow suppression or late-stage infection where eosinophil recovery begins.

Supporting evidence from literature

Multiple studies reinforce the diagnostic role of eosinopenia in typhoid fever. Gandhi et al. [12] observed eosinopenia in 42 of 45 culture-positive cases. Matono et al. [22] reported absolute eosinopenia in 63% of returning travelers with enteric fever, and a negative predictive value of 83-92%. These findings strengthen the use of eosinopenia as a decision-support tool in triaging patients with febrile illness, particularly in low-resource settings. While these studies vary in design and thresholds, the consistency of trends across different populations adds confidence to the marker’s value.

Pediatric considerations and age-based variability

Eosinopenia may be especially useful in pediatric populations. Gupta et al. [18] found it in 81.2% of children with enteric fever. Chitkara et al. [23] also noted its higher prevalence in pediatric versus adult patients. Although we did not stratify diagnostic performance by age in this study, these results support the potential of eosinopenia as a practical clue in children with atypical presentations. Future studies should explore age-stratified thresholds to enhance specificity.

Clinical utility and study limitations

This study adds quantitative evidence that absolute eosinopenia is a specific diagnostic clue in culture-confirmed enteric fever. Its use may be especially helpful in primary and secondary care settings where blood culture facilities are limited or delayed. However, it should not be used in isolation. Clinical history, epidemiological exposure, and other laboratory findings (e.g., leukocyte counts) must be integrated. The retrospective design limits causal inference, and the absence of multivariate adjustment may have introduced unmeasured confounding. Additionally, as a single-center study, external generalizability is limited. We also did not stratify by pathogen (S. Typhi vs. S. Paratyphi), nor examine eosinophil recovery kinetics.

Summary of clinical implications

Absolute eosinopenia is a rapid, cost-effective, and highly specific marker that may aid early diagnosis of enteric fever. When used alongside leukocyte counts and clinical judgment, it can improve diagnostic confidence and guide empirical treatment decisions, especially in resource-constrained or endemic settings. Future prospective studies should evaluate eosinopenia’s role in diagnostic algorithms and its integration into risk-scoring tools.

## Conclusions

Although absolute eosinopenia has previously been associated with enteric fever, this study reinforces its diagnostic value by providing region-specific, statistically validated evidence from a well-defined case-control cohort. Absolute eosinopenia demonstrated high specificity, supporting its role as a rapid adjunctive marker for enteric fever diagnosis-particularly in resource-limited settings where serological testing may not be readily available. However, its moderate sensitivity limits its standalone diagnostic utility. Clinicians should therefore interpret eosinopenia in conjunction with leukocyte counts and relevant clinical features to improve diagnostic decision-making. While this study did not directly assess additive effects of combined laboratory parameters, existing literature suggests that a multi-marker approach may enhance early diagnostic accuracy. Future research should focus on refining eosinopenia-based thresholds, developing diagnostic algorithms incorporating hematological markers, and validating these tools across diverse populations through prospective, multi-center studies.
